# Aphid Wing Induction and Ecological Costs of Alarm Pheromone Emission under Field Conditions

**DOI:** 10.1371/journal.pone.0011188

**Published:** 2010-06-23

**Authors:** Eduardo Hatano, Grit Kunert, Wolfgang W. Weisser

**Affiliations:** 1 Institute of Ecology, Friedrich-Schiller University Jena, Jena, Germany; 2 Max Planck Institute for Chemical Ecology, Jena, Germany; Michigan State University, United States of America

## Abstract

The pea aphid, *Acyrthosiphon pisum* Harris, (Homoptera: Aphididae) releases the volatile sesquiterpene (*E*)-β-farnesene (EBF) when attacked by a predator, triggering escape responses in the aphid colony. Recently, it was shown that this alarm pheromone also mediates the production of the winged dispersal morph under laboratory conditions. The present work tested the wing-inducing effect of EBF under field conditions. Aphid colonies were exposed to two treatments (control and EBF) and tested in two different environmental conditions (field and laboratory). As in previous experiments aphids produced higher proportion of winged morphs among their offspring when exposed to EBF in the laboratory but even under field conditions the proportion of winged offspring was higher after EBF application (6.84±0.98%) compared to the hexane control (1.54±0.25%). In the field, the proportion of adult aphids found on the plant at the end of the experiment was lower in the EBF treatment (58.1±5.5%) than in the control (66.9±4.6%), in contrast to the climate chamber test where the numbers of adult aphids found on the plant at the end of the experiment were, in both treatments, similar to the numbers put on the plant initially. Our results show that the role of EBF in aphid wing induction is also apparent under field conditions and they may indicate a potential cost of EBF emission. They also emphasize the importance of investigating the ecological role of induced defences under field conditions.

## Introduction

Aphids are important economic insects in temperate regions, damaging plants by sucking nutrients from the phloem and transmitting plant viruses [1], [2]. Because of their abundance, aphids are attacked by a wide range of predators such as ladybirds, lacewings and hoverfly larvae, all of which showed to influence strongly the growth and persistence of aphid colonies [3].

In response to a predator direct attack, aphids secrete cornicle droplets from a pair of tube-like structures on the abdomen called siphunculi [4]–[6]. The droplets glue together the predator's mouthparts [4], and in addition, they contain an alarm pheromone, the sesquiterpene (*E*)-β-farnesene (EBF), which is for some aphid species the main or only pheromone compound present [6]–[11]. EBF triggers various behavioural reactions in aphids, like withdrawing the stylets from the plant, or dropping off their host plants [12], [13]. EBF may also attract some species of aphid predators [14]–[16] and parasitoids [17] and might be used by plants to deter aphids [18].

Polyphenism is one of the main characteristics of aphids and during the phase of asexual production in summer, both winged and unwinged females occur. In the case of the pea aphid, *Acyrthosiphon pisum* Harris (Homoptera: Aphididae), wing formation among offspring is maternally induced when the mother is under adverse biotic conditions, for example, triggered by crowding, low host plant quality, or the presence of natural enemies [19]–[28]. Recently, EBF was also found to mediate indirectly the production of winged offspring of the pea aphid [29], by increasing the number of tactile stimuli among individuals of a colony (pseudo crowding effect) [23], [29]. This effect is analogous to the response of aphids to an increasing colony size (crowding), when the number of tactile interactions also increases [26]. While predator-induced wing formation in pea aphids [19], [20], [24], [28], [30] and its mediation by EBF [29] were repeatedly demonstrated in the laboratory, the importance under natural conditions has so far not been investigated. It is conceivable that air movements change the amount and/or concentration of detectable EBF in an aphid colony, possibly alerting fewer aphids than under laboratory conditions. In addition, many pea aphids that perceive EBF walk away from the original plant and often do not survive during migration because of starvation or ground predators [31]. Both effects decrease the population density on the plant and, consequently, may weaken the pseudo crowding effect and the production of winged morphs. Furthermore, the aphid alarm pheromone can act as kairomone by attracting natural enemies [32], and predation would further lower the number of aphids in a colony and also reduce the pseudo crowding effect [20].

In the current study, we tested the hypothesis that pea aphids under field conditions also produce higher proportion of winged offspring after reacting to EBF like observed in laboratory experiments. Our objective was to determine the role of EBF for wing induction and aphid fitness under field conditions and to compare it to a laboratory test. To do this, we exposed colonies of pea aphids daily to the alarm pheromone under field and laboratory conditions, and scored the proportion of winged offspring and the number of individuals on the plants at the end of experiment.

## Materials and Methods

### Plant and aphid material

Pink pea aphids of clone BP [29] were reared on 3-week-old broad bean plants, *Vicia faba* L (variety The Sutton; Nickerson-Zwaan, UK). Plants were cultured in pots (10 cm diameter, 8 cm high) and covered with air-permeable bags (L×W = 39×20 cm, Armin Zeller, Nachf. Schütz & Co, Langenthal, Switzerland) to avoid aphid escape. Infested plants were kept in the climate chamber under constant conditions (16∶8 L:D; 20°C; 75% RH).

### Aphid lines

Twenty-eight aphid lines were set up as described by Kunert et al. [29]. One aphid line consisted of the genetically identical progeny of a single aphid. For one line, one adult aphid was first placed on a three-week-old broad bean plant, allowed to reproduce for 48 hr, and then removed from the plant. After nine days, the daughters (10 aphids per line), now adults, were transferred to five new plants (two aphids per plant) to avoid crowding. After 48 hr reproducing, the daughters were removed, leaving twelve granddaughters per plant. After another six days, the granddaughters became third- and fourth-instar nymphs and sixty aphids from each line were transferred to four new broad bean plants in groups of fifteen aphids. The four plants per line were randomly allocated to one of four treatments (see below). In this way, both maternal effects and any effects of the plants on which aphids were reared were distributed equally over all treatments.

### Experimental design

We tested the effect of EBF on aphid wing induction by exposing aphid colonies to either artificial EBF (EBF treatment) or a solvent control (control treatment) three times per day for five days. The experiment was set up simultaneously in two locations: in the field and in the climate chamber, resulting in a 2 (pheromone application) ×2 (location) factorial design.

#### Field test

Pairs of plants with aphids (granddaughters) from the same line were placed at a distance of five metres from one another and ten metres between pairs along the margins of the Jena biodiversity field experiment [33] in Jena. The daily means of temperature ranged from 17.4°C to 20.3°C, relative humidity ranged from 75.9% to 88.2%, precipitation ranged from 0.007 mm to 0.566 mm, and wind speed ranged from 0.8 m/s to 21.2 m/s over the 5-day experimental period. One plant in each pair was allocated to the EBF treatment, the other one to the control. A toothpick holding a square piece (1×1 cm) of filter paper was fixed inside each pot in the soil. To reduce the access of natural enemies to aphid colonies, all plants were enclosed by cages, 30 cm in height, made from aluminium mesh (mesh width, 2 mm) fixed using adhesive to a plastic frame of a plant saucer (25 cm i.d.) from which the bottom was removed. Cages were sprayed with insect glue (Soveurode, Witasek) and the bottom edges were pressed into the ground and covered with soil to prevent predators or other insects to enter the cages.

For five days, 5 µl of EBF solution containing 1000 ng EBF (0.20 µg EBF per 1 µl hexane; EBF treatment) or 5 µl hexane (control) were applied three times a day (at 8:00, 13:00 and 18:00) onto the filter paper of each pot through the mesh of the cages using a micropipette. The amount of EBF applied was enough to be perceived by the aphids and to elicit the alarm behaviour under field conditions. In addition, this amount was also used by Kunert et al. [29] who discernibly showed that the frequency of EBF emission per day rather than amount of EBF emitted regulates the proportion of wing offspring produced.

After five days, the adult aphids on the broad bean plants were counted and removed. Plants with aphids were covered with cellophane bags and transferred to the climate chamber with same conditions described above and kept until all nymphs became L4/adults. When offspring had reached maturity, all aphids from each plant were removed from the plant and frozen at -18°C, after which offspring number and offspring phenotype were counted.

#### Climate chamber test

The second pair of infested broad bean plants from each line was kept under climate chamber conditions (16∶8 L:D; 20°C; 75% RH) as a positive control. Plants were covered with cellophane bags so aphids could not escape. EBF was applied and aphids were handled exactly as in the field.

### Statistical Analysis

All analyses were carried out with the R software version 2.8.1. The number of adult aphids found on plants after the experiment and the number of offspring produced were analysed using generalized linear models (GLM). Because overdispersion was detected during analysis, a quasibinomial (for proportion of aphids found on the plants) and quasipoisson (for offspring count data) error structures were used in our analyses [34], [35]. Because of non-normality of the data, proportions of winged morphs were square root transformed and analysed by an ANCOVA, using the total number of offspring as a covariate. In all models, aphid lines were included as a random effect.

Models were simplified by reducing non-significant interactions followed by independent variables that were not included in any significant interaction [36]. Among non-significant independent variables or interactions with same number of variables, the one with highest p value was first removed followed by others in a descending order. After removing a non-significant interaction or variable, a new model was generated and only accepted if the removal did not significantly increase deviance comparing to the previous model after a *F* test (*p*>0.05) [37]. Otherwise, the previous model was retained and the simplification continued with the next non-significant interaction or variable. When an interaction of variables was found significant, the corresponding levels were compared using contrasts [36]. Results are presented as mean ± SE.

## Results

### Proportion of adult aphids found on plants at end of experiment

**Figure 1:**
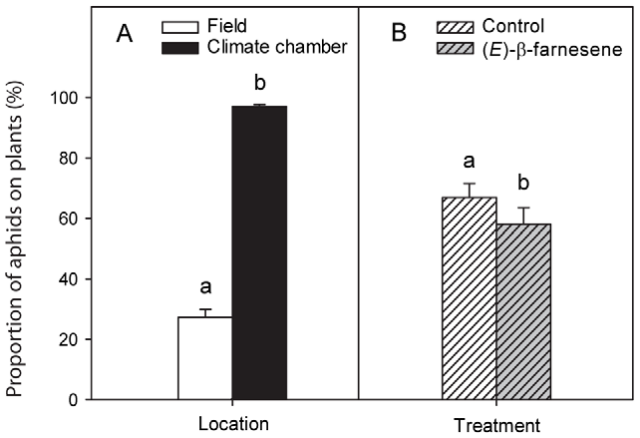
Proportion of adult pea aphids found on the plant at the end of the experiment. Aphids were either exposed to A) alarm pheromone and control (left), and B) under field and climate chamber conditions (right). Initially, 15 aphids were introduced to each plant and the proportions of remaining adult aphids were recorded after five days in the field and in the climate chamber, for both the EBF (black bars) and control (white bars) treatments. The bars show mean values + SE.

One replicate of the field control treatment was removed after the first day of experiment because no aphid was found on this plant. In the laboratory, the proportion of adult aphids (granddaughters) that were found on the plants at the end of experiment was very high (97.02±0.72%) regardless of the pheromone treatment, i.e. on average less than one aphid died over the five-day experimental period. In contrast, this proportion was much lower in the field where on average less than a third of the 15 aphids were found back on the plant (27.27±2.68%, *t*_108_ = 13.939, *p*<0.001, Fig. 1A).

The application of alarm pheromone resulted in a significant lower proportion of adult aphids found on the plant at the end of the experiment (58.09±5.50%) compared to the control (66.90±4.59%, *t*_108_ = 3.331, *p*<0.01, Fig. 1B). Although there was no interaction between the experiment location and pheromone treatment (*F*_1,108_ = 2.22, *p* = 0.13), we compared the proportion of mothers found on the plant in the different treatments separately for both locations to investigate the possible negative effects of EBF for aphids. For this purpose, we performed the same GLM test with a quasibinomial error distribution using orthogonal contrasts [38]. In the climate chamber, the numbers of adult aphids found on the plants at the end of the experiment did not differ between control and EBF treatments (*t*_55_ = 8.144, *p* = 0.766). However, in the field, the numbers of aphids found on the plant at the end of experiment were on average only 55% of the corresponding numbers in the control treatment, i.e. they were significantly lower (*t*_53_ = 4.134, *p*<0.001). Although cages protected the plants from natural enemies, some ants were observed in few cages at the end of experiment.

### Total number of offspring

**Figure 2:**
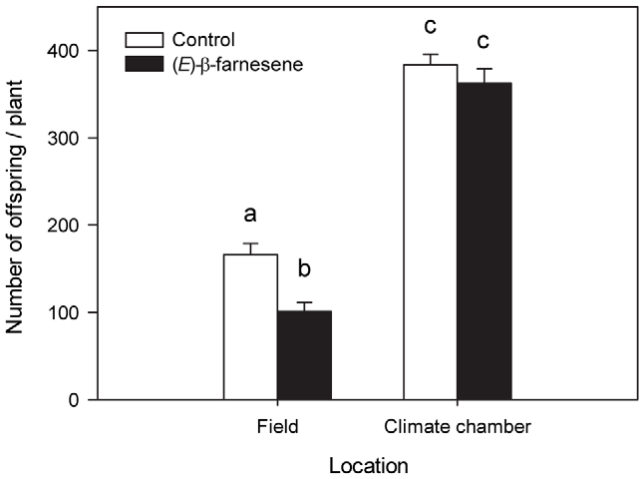
Colony sizes of aphids exposed to alarm pheromone and control under different conditions at the end of the experiment. Offspring on each plant were counted after five days of experiment in the field and in the climate chamber, when aphid colonies were treated with either control (white bars) or (E)-β-farnesene (black bars) (F_1,107_ = 11.969, p<0.001). Bars with different letters are statistically significant different (P<0.001). The bars show mean values+SE.

In total, 28273 offspring were counted in the experiment. Significantly more offspring were recorded in the climate chamber than in the field (*t*_107_ = 10.102, *p*<0.001), and more offspring were born in the control than in the EBF treatment (*t*_107_ = 4.414; *p*<0.001). The interaction between location and pheromone application was significant (*F*_1,107_ = 11.969, *p*<0.001), i.e. the difference between control and EBF treatment was dependent on where the experiment was carried out: a significant difference between EBF and control was observed under field conditions (*t*_53_ = 76.862, *p*<0.001; Fig. 2) but not under climate chamber conditions (*t*_55_ = 0.750, *p* = 0.455; Fig. 2).

### Offspring phenotype

**Figure 3:**
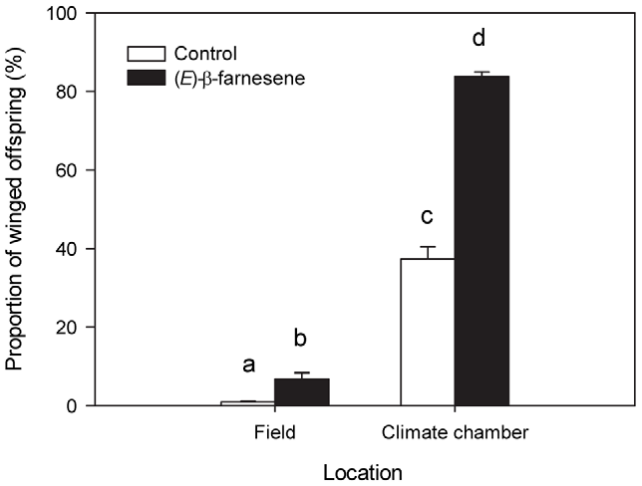
Induction of wing formation in offspring from colonies exposed to alarm pheromone and control under different conditions. The proportions of winged morphs among offspring were recorded in the field and in the climate chamber, for both the control (white bars) and (E)-β-farnesene (black bars) treatments (F^1,103^ = 38.784, p<0.001). Bars with different letters are statistically significant different (p<0.01). The bars show mean values+SE.

Whilst the proportion of winged morphs among the offspring was higher in the climate chamber compared to the field (*t*_103_ = 1.113; *p*<0.001), the application of EBF significantly increased wing induction (*t*_103_ = 1.138; *p*<0.001, Fig. 3). The interaction between location and pheromone application was also significant (*F*_1,103_ = 38.784, *p*<0.001, Fig. 3). In the climate chamber, the proportion of winged morphs among the offspring was on average 124% higher in the EBF treatment than in the control (*t*_55_ = 10.444, *p*<0.001, Fig. 3). In the field, the proportion of winged offspring increased by 600% from the control to the EBF treatment (*t*_54_ = 2.786, *p*<0.01, Fig. 3).

**Figure 4:**
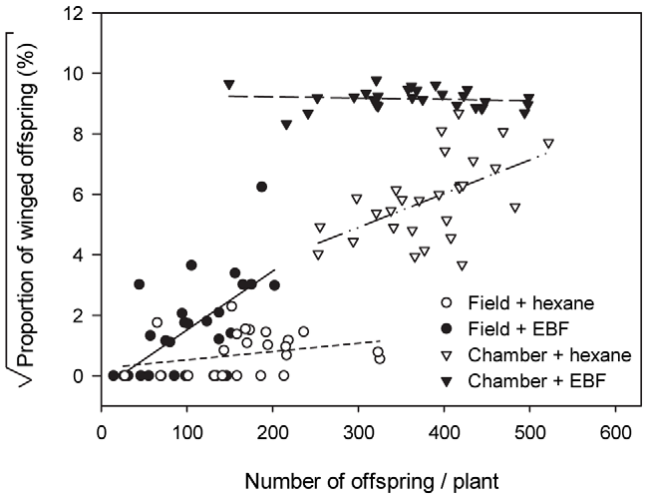
Wing induction of offspring in different colony sizes exposed to alarm pheromone and control under different conditions. The square root transformed proportion of winged offspring as a function of the number of offspring in the field and in the climate chamber, treated with either EBF or hexane. White circles represent field colonies treated with hexane control (0.2679+0.0025X, r^2^ = 0.058, F_1,25_ = 1.525, p = 0.228); black circles are field colonies treated with EBF (−0.420+0.019X, r^2^ = 0.448, F1,26 = 21.08, p<0.01); white triangles are chamber colonies treated with hexane control (1.920+0.011X, r2 = 0.209, F_1,26_ = 7.146, p = 0.0126); and black triangles are chamber colonies treated with EBF (9.306-0.00041X, r^2^ = 0.011, F_1,26_ = 0.288, p = 0.596).

The interaction among location, pheromone application and number of offspring was also significant (*F*_2,103_ = 13.788; *p*<0.001, Fig. 4): in the field, the proportion of winged offspring was not correlated to the number of offspring in the control treatment (0.2679+0.0025X, *r* ^2^ = 0.058, *F*_1,25_ = 1.525, *p* = 0.228, Fig. 4), while there was a positive correlation in the EBF treatment (−0.420+0.019X, *r* ^2^ = 0.448, *F*_1,26_ = 21.08, *p*<0.01, Fig. 4). Under climate chamber conditions, the opposite was observed: the number of offspring positively affected the proportion of winged morphs in the control (1.920+0.011X, *r* ^2^ = 0.209, *F*_1,26_ = 7.146, *p* = 0.0126, Fig. 4), but did not in the EBF treatment (9.306-0.00041X, *r* ^2^ = 0.011, *F*_1,26_ = 0.288, *p* = 0.596, Fig. 4).

## Discussion

While laboratory experiments are an important tool in revealing ecological mechanisms, field experiments are needed to test the ecological relevance of the observed effects. Our results show for the first time that EBF mediates the production of winged pea aphid offspring along with colony size under field conditions. In addition, our experiment showed that the proportions of adult aphids found on the plants at the end of the experiment was not only lower in the field than in the climate chamber (Fig. 1A), but it was also negatively affected by the application of EBF (Fig. 1B), resulting in fewer offspring than in the hexane control (Fig. 2). While no dead aphid bodies were recovered at the end of the experiment, it is most likely that aphids not found at the experiment died in the course of the experiment, i.e. the number of aphids found on the plant is a measure of aphid survival (see also below).

Pea aphids trigger the production of winged morphs when in repeated physical contact with each other, as in the case of high aphid densities on a plant, which indicates high intraspecific competition levels (crowding effects; [26]). Therefore, smaller colonies are less likely to produce winged morphs than larger ones because of less physical contact between colony members [26]. Yet the proportion of dispersal morphs was higher in the EBF treatment, even though only 2.9±0.5 adults remained on the EBF treated plant compared to plants treated with hexane in which 5.2±0.6 adults remained (Fig. 1 and 3). When aphid colonies are exposed to EBF in laboratory conditions, the proportion of winged offspring increased with the initial number of aphids on a plant [39].

The climate chamber data reported here are very similar to those of the aphid group size of 13 in Kunert et al. [39]. In contrast, Kunert et al. [39] reported winged offspring production of 10% (control) and ca. 40% (EBF treatment) when initial aphid number was two. In our field experiment, the number of adult aphids put on the plant initially in our field test was higher than two; the percentage of winged offspring observed was lower than what was observed in Kunert et al.'s experiment. This indicates that wing induction in the field is reduced not only by lower number of mothers (Fig. 1) but also by other factors. Airflow in the field very likely reduces the amount and concentration of EBF that reaches aphids, such that possibly only aphids near the source perceive biologically relevant amounts of EBF, resulting in a general decrease in the response.

The increase of produced offspring enforced the pseudo crowding and crowding effects on each plant when EBF or control hexane was applied, respectively, and, therefore, also played a positive role in wing induction (cf. [20], Fig. 4). In addition, the large cages in the field test allow aphids to walk off the host plant and this might reduce the contact rate among individuals compared to the smaller cellophane bags in the climate chamber, where aphids leaving the plant are likely to return to it immediately. Finally, while the same aphid clone and the same plant species was used in the present experiment and in the experiments of Kunert et al. [29], small differences in manipulation may also have influenced the response of the experimental aphids towards the wing-inducing cues.

In the laboratory, there was no effect of EBF on the number of adult aphids found on the plant at the end of the experiment, indicating that the concentrations of EBF or hexane applied were not toxic to the pea aphids. Both, the location where the experiment was carried out, and the solution applied, independently affected the proportion of aphids that were found on plants at the end of the experiment. While in the laboratory aphids enclosed in cellophane bags could not move away far away from their plants and therefore were likely to find the plant again after leaving it, aphids in larger field cages were likely to spend more time searching for their hosts, increasing the possibility for desiccation or starvation and resulting in an overall decrease in fecundity [40]–[42].

A significant reduction in the number of adults on plants treated with EBF was also made by Wohlers [31] who reported that when pea aphids were dislodged by exposure to synthetic EBF they moved towards neighbouring plant models while a small proportion of aphids climbed back to the original plant. By making use of the alarm signalling behaviour, Bruce et al. [43] successfully reduced the aphid population in field plots using plant extracts containing 70% EBF and a slow-release point sources which probably resembled the natural emission of EBF from aphids [44].

An additional cost of the alarm pheromone perception might be the higher predation risk of aphids which left the plant [45]. Although the plants in the field were protected with cages, ants were able to enter the cages from below; hence it is likely that not only starvation but also predation contributed to the observed decrease the numbers of aphids in the field. A relationship between aphid alarm pheromone and ant aggression was reported before. In a comprehensive study, Nault et al. [46] exposed several myrmecophilous and non-myrmecophilous aphid species in a laboratory setting to ants, predators and alarm pheromone. Ants near myrmecophilic aphids became very aggressive in the presence of EBF and increased their rate of attack on aphid predators, but they did not attack aphids. However, when an alarm pheromone was applied to colonies of untended aphid species, ants became aggressive towards the aphids and sometimes carried them off the plant [46]. Similar observations of aggressive behaviour of aphid-attending ants towards an EBF source were made in the field [47], [48].

Costs of alarm signalling was recently discussed by Verheggen et al. [49], who demonstrated that pea aphids regulate the emission of EBF according to social environment, with small colonies releasing less EBF than large colonies. In this context, aphids reduce the predation risk by not attracting natural enemies and remaining inconspicuous while they reduce physiological cost to produce EBF.

In conclusion, our study shows that EBF mediates wing induction in pea aphid colonies not only under laboratory but also under natural conditions. The experiment under natural conditions also pointed to the importance of colony size in interaction with alarm signalling to produce winged offspring by the pseudo crowding effect. Now since we know that wing induction in aphids also occur under natural conditions it is important to investigate whether there is an ecological cost involved in alarm pheromone emission in detail.
